# Novel biomarkers for potential risk stratification of drug induced liver injury (DILI): A narrative perspective on current trends

**DOI:** 10.1097/MD.0000000000018322

**Published:** 2019-12-16

**Authors:** Mohammed Ibn-Mas’ud Danjuma, Jamal Sajid, Haajra Fatima, Abdel-Naser Elzouki

**Affiliations:** a Hamad General Hospital, Hamad Medical Corporation (HMC), College of Medicine Qatar University; b Hamad General Hospital, Hamad Medical Corporation (HMC); c Internal Medicine Residency Program, Hamad Medical Corporation; d Hamad General Hospital, Hamad Medical Corporation (HMC), Doha, Qatar.

**Keywords:** biomarker, DILI, liver, transaminases

## Abstract

**Background:**

Drug induced liver injury (DILI) is an increasing cause of acute liver injury especially with increasing need for pharmacotherapy of widening comorbidities amongst our ever-aging population. Uncertainty however remains regarding both acceptable and widely agreeable diagnostic algorithms as well a clear understanding of mechanistic insights that most accurately underpins it. In this review, we have explored the potential role of emerging novel markers of DILI and how they could possibly be integrated into clinical care of patients.

**Methods:**

We explored PUBMED and all other relevant databases for scientific studies that explored potential utility of novel biomarkers of DILI, and subsequently carried out a narrative synthesis of this data. As this is a narrative review with no recourse to patient identifiable information, no ethics committee's approval was sought or required.

**Results:**

Novel biomarkers such as microRNA-122 (miR-122) profiles, high mobility group box-1 (HMGB1), glutamate dehydrogenase (GLDH), and cytokeratin-18 (K-18), amongst others do have the potential for reducing diagnostic uncertainties associated with DILI.

**Conclusion:**

With the increasing validation of some of the novel liver biomarkers such as K-18, mir-122, HMGB-1, and GLDH, there is the potential for improvement in the diagnostic uncertainty commonly associated with cases of DILI.

## 1 Introduction

Drug induced liver injury (DILI) is a fast growing area of both clinical and therapeutic concerns.^[1]^ This is due to a number of factors including increasing patient survival with increasing morbidities and need for varied pharmacotherapy, unresolved uncertainties regarding the exact diagnostic algorithm of DILI, as well unreliability of current biochemical markers and causality assessment tools in adjudicating suspected cases of DILI. Despite its rarity (<1% from most patient series), it has been estimated to account for the most common cause of acute liver failure in both Europe and United State.^[2]^ The incidence of clinically significant DILI varies from country to country. In France for example, its annualized incidence stands at about 14 to 19 cases per 100,000 population.^[3]^ It is similarly a significant cause of drug development attrition in the course of potential candidate drug evaluations in early phase studies and therefore failure to reach the market.^[4]^ DILI is said to be intrinsic if the observed effect is demonstrably dose dependent. This is the case with rise in serum transaminases (ALT 3X the upper limit of normal {ULN}) seen in a proportion of patients following exposure to Acetaminophen at its recommended dose (4 g/day) for about 2 weeks.^[5]^ However, in about 10% to 15% of DILI, the cause, dose, and temporal association with incriminating drug(s) is less clear. This is rare and called idiopathic DILI (IDILI). Unlike DILI, in these cases, a clear dose dependency cannot be established, and latency between drug exposure and transaminase rise sometimes extending up days, weeks, or even in some cases months. Whilst the mechanism for IDILI is still a matter for mechanistic debate, current evidence suggests that drug-protein adducts formed following exposure to certain drugs are taken up by immune competent cells, processed and presented as new antigens.^[2]^ The consequence of this is the ensuing immunoallergic reaction with varying presentation as various clinical/biochemical liver-related morbidities including transaminase rise. Prior hepatic injury from a wide variety of sources including infections (viral hepatitis), autoimmune hepatitis amongst others appears to increase the susceptibility to this type of injury.^[6]^ Why only a designated cohort of patients develops this may probably be a factor of possession of single nucleotide polymorphisms of genes encoding proteins involved the biodisposition of these drugs.^[3]^ In this review we have narratively appraised current evidenced as it relates to proposed biomarkers of DILI with the view to ascertaining their accuracy in determining cases of suspected DILI. As this is a narrative review with no recourse to patient identifiable information, no ethics committee's approval was sought or required.

## 2 What are current traditional markers of DILI, and are there distinct diagnostic phenotypes?

At presentation there is no agreement as to any distinct diagnostic clinical phenotype of DILI. Concomitant presence of associated skin dermatosis and rash is sometimes clinically used to denote possible DILI as the probable diagnosis.^[7]^ Until recently, the kinetics of liver transaminases such as alanine aminotransferase (ALT), and aspartate aminotransferase (AST) in common with total bilirubin (TBL) and alkaline phosphatase (ALP) have been utilized in both clinical and, clinical trial settings for the determination of suspected cases of DILI.^[8]^ The intracellular nature of AST and ALT within hepatocytes meant that a sudden rise in their levels and changes in their plasma kinetics denotes hepatocellular injury, and may correlate with proportion of hepatocellular loss. The cause of this injury is however non-specific and may range from drugs, infections, inflammatory disorders, toxins, to autoimmune insults amongst others. ALP on the other hand is more associated with canalicular membrane or biliary epithelial cells, with rise in its level denoting injury to these sites or intra/extra hepatic obstruction of biliary tracts. Elevated levels of TBL are none specific as a discriminant of hepatic injury as a rise in its kinetics is factor of either increased production from intravascular hemolysis, or altered processing by the liver. It is therefore evident from the above that utilized on their own, these age-long markers of “liver injury” are limited in both their ability to establish the relevant etiology of such injury (ie, DILI or otherwise), or offer any reasonable insight into the mechanism (s) underlying the mode of injury. Additionally, the delayed nature of their elevation vis-à-vis establishment of definite cases of DILI in particular negates their value as reliable and early markers of this ever-increasing morbidity. More so the evident lack of reliable correlation between magnitude of enzyme elevation and severity of hepatocyte injury meant that a dependable and robust prognostication could not be made.^[9]^

That notwithstanding, there have been attempts at employing specific diagnostic algorithms, utilizing temporal profiles of a combination of these transaminases in order to determine DILI as cause of some patient presentations. Amongst the recently developed and widely used causality assessment tools includes Roussel-Uclaf Causality Assessment Method (RUCAM), and a consensus based expert opinion approach amongst others.^[10]^ Additionally, other risk stratification and diagnosis adjudicating algorithms such as R-value evaluates the ratio of ALT to ALP at presentation in order to make a determination as to whether DILI was primarily hepatocellular, cholestatic, or mixed.^[4,5]^ An R-value of >5 were reported as been in tandem with hepatocellular injury, a value <2.5 support the diagnosis of cholestasis as the cause of DILI, whilst a value raging between 2.5 and 5 denotes a mixed picture.^[4,5]^ How substitution of ALT for AST in the R equation enhances the discriminant power of this equation has continued to generate intense statistical debate.^[6]^ These causality assessment tools are limited by the same limitation of the variables they incorporate (ALT, AST, ALP, TBL) in various combinations for DILI case ascertainment. In a further attempt to minimize confounding of DILI case ascertainment particularly in clinical trial environments, the food and drug administration (FDA) issued the *Hy law* to guide and increase the diagnostic accuracy of bilirubin and serum transaminases. This law defines a typical *Hy* law case as the event that most accurately predicts the risk of Liver failure.^[11]^ In further clarification of this guidance the FDA in 2009 advised that a typical *Hy* law case is a patient with normal Liver at the commencement of a clinical trial, but who then develops elevation in ALT or AST 3X ULN and associated rise in serum TBL of 2X ULN with no discernible cause for this other than the offending drug.^[12]^ Subsequent validation of this law/observation by analyses of various registry data around the world showed that up to 10% of patients with drug induced hepatocellular jaundice go on to develop liver failure.^[12]^Table 1 gives a comparative summary of current clinico-laboratory parameters, limitations, and algorithms for DILI adjudication.

**Table 1:**
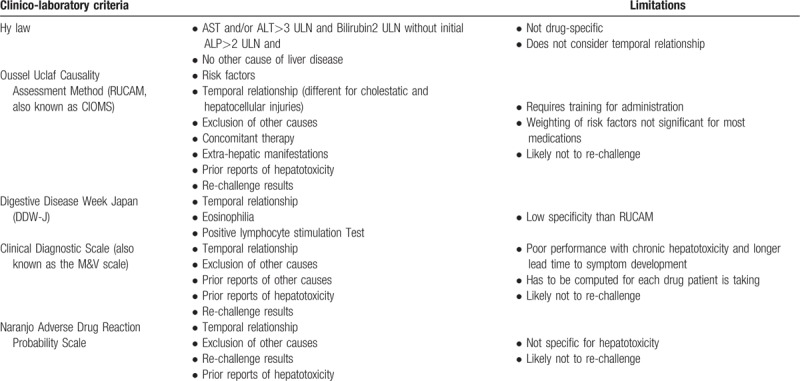
Current Clinico-laboratory criteria for DILI adjudication including variables utilized and evident limitations.

In light of the afore-mentioned limitations of liver enzymes as diagnostic markers of DILI as well as the ever-increasing morbidity of this problem, there is a compelling need to develop DILI-specific biomarkers that add both diagnostic and prognostic insight into this problem. But additionally, help explain some or all of the mechanistic processes behind the development of DILI.

## 3 What is the current “state of play” regarding the role of liver biopsy in the adjudication of DILI?

Liver biopsy very often occupies a final adjudication point in the diagnostic evaluation of liver injuries or pathologies from a wide variety of etiologies.^[13]^ In DILI however, the lack of diagnostic clarity seen with common laboratory assays and clinical algorithms unfortunately extends to liver biopsy as well.^[13]^ No histopathological feature is pathognomonic of DILI.^[14,15]^ This is so because known and already reported histopathological patterns associated with DILI mirrors a wide variety of lesional liver injury patterns from disparate and unrelated etiologies. Despite these limitations however, sometimes correlation of liver injury with the patient's medication history, and some salient clinical features may assist in narrowing down the possible differential diagnoses.^[13]^ This is particularly so when it is recognized that most drugs have a limited range of histological features and vary in their propensity to cause injury.^[13–16]^ Unlike more typical features of common pathologies such as chronic hepatitis and fatty liver disease, biopsy of suspected DILI does show a wide variety of histopathological features such as inflammation, necrosis, cholestasis, fibrosis, nodular regeneration, vascular injury, duct destruction, and granuloma among others.^[14]^ In Hans Popper's seminal report on drug and toxin induced liver injury, acute viral hepatitis-like injury and cholestatic hepatitis accounted for 39% and 32% of the cases, respectively.^[16]^ A more recent analysis of 249 liver biopsies of suspected DILIs found that over half of them could be classified into one of six necro-inflammatory and cholestatic injury patterns.^[13]^ These patterns include cholestatic hepatitis (29%), acute hepatitis (21%), chronic hepatitis (14%), chronic cholestasis (10%), acute cholestasis (9%), and zonal necrosis (typical pattern of acetaminophen DILI) (3%). There have been efforts to relate the histopathological features with distinct clinical severity of liver disease.^[13]^ Varying degrees of necrosis and presence of ductular reaction correlates with liver transplant and death. Other patterns such as hepatic necrosis, fibrosis, microvesicular steatosis, cholangiolar cholestasis, neutrophils, and portal venopathy were associated with either severe, or fatal injury. Although not invariable, hepatic granulomas were associated with mild or moderate liver injury.^[13]^ Indeed this lack of reliable correlation between hepatic histopathology phenotypes and DILI has resulted in the lack of inclusion of liver biopsy in DILI adjudication algorithms including RUCAM algorithm.^[10]^

**Figure 1:**
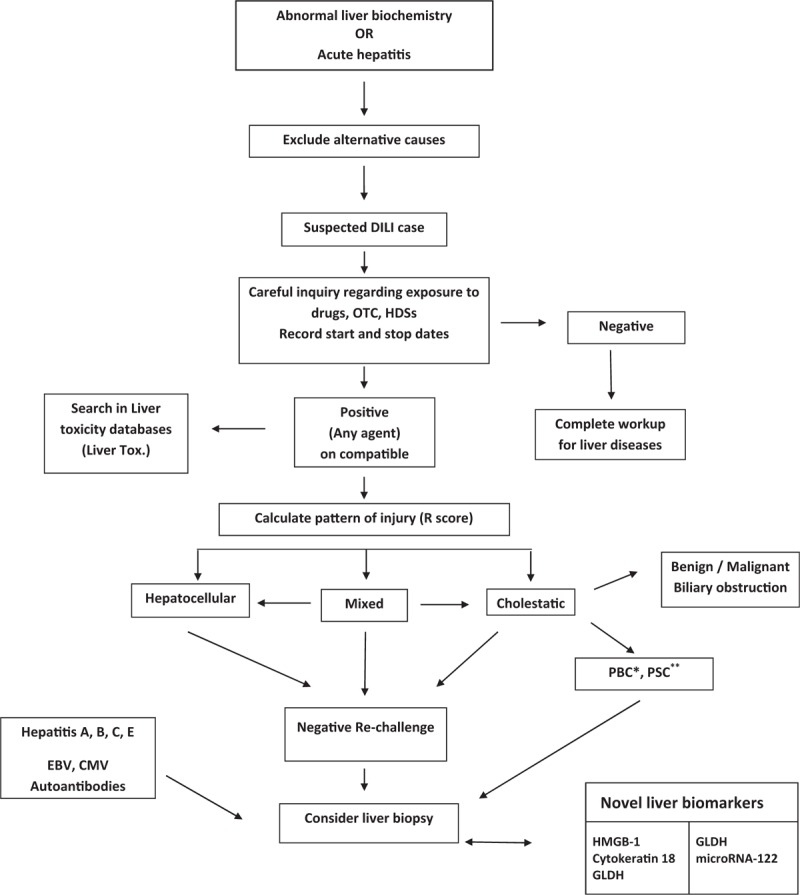
Diagnostic algorithm for suspected presentation with drug-induced Liver injury (DILI). A schematic representation of the proposed role of novel liver biomakers in diagnostic alrightm of DILI, and how they fit into current diagnostic pathways.

From the foregoing it is evident that liver biopsy does not provide a conclusive pathognomonic tool for a determinative diagnosis of cases of DILI, as no hepatic histopathological feature is pathognomonic of DILI. This has to do with the myriad of histopathological phenotypes associated with DILI, depending on the individual offending drug (s). Despite this however, Liver biopsy still remains the ultimate arbiter or gold standard in clinical use today where uncertainty arises as regards etiology of acute liver injury including cases of DILI. Histopathological results/phenotypes can only be used in addition to clinical features and biochemical profiles to assist in both diagnosis and adjudication of suspected cases of DILI. Figure 1 shows a proposed diagnostic algorithm for a suspected case of DILI.

## 4 What are the novel markers of DILI currently in development?

Since the evolution of the current system-based assessment of both therapeutics and diagnostic protein targets (“omics”), there has been tremendous interest in targeting proteins and their metabolites. These include DNA/RNA, and their gene products with the view to determining any potential “toxicity signatures” that are likely to correlate with DILI and enhance current understanding of the pathophysiological processes.^[18]^ Metabolomics in particular provides a very high throughput platform (by mass spectrophotometry for evaluation of these potential liver toxicity markers.^[18]^

As the search for reliable candidate markers of DILI continues, initial results indicate that there is the lack of homogeneity in both the pharmacokinetics and pharmacodynamics of the offending drugs that cause DILI. This therefore has meant that initial efforts have targeted the drug most prevalently associated with DILI,^[12,18]^ The totality of current evidence suggests that over half of all DILI related cases are cause or associated with Acetaminophen,^[5,9,20]^ The later perhaps explains why recent efforts at DILI biomarker search have focused on Acetaminophen-induced liver injury. Furthermore, a significant proportion of both clinical and laboratory data from recent work on DILI biomarkers have largely been on Acetaminophen.^[9]^Table 2 gives a comparative summary of diagnostic/prognostic characteristics of novel liver biomarkers currently in development.

**Table 2:**
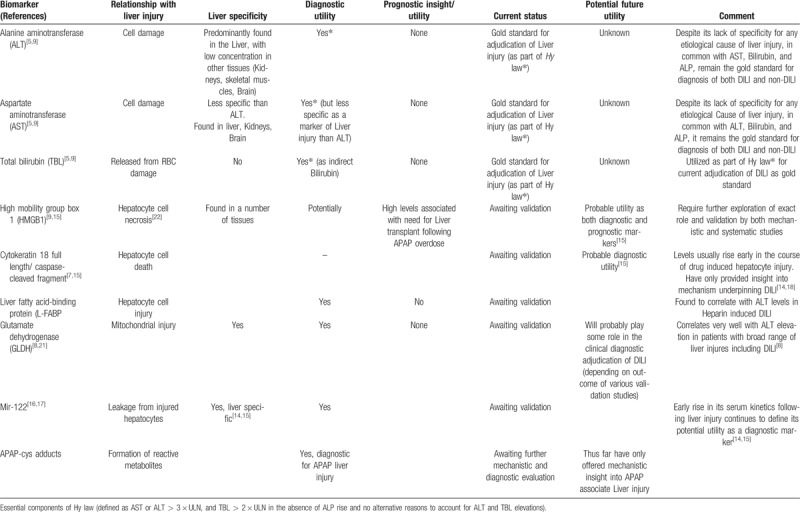
A comparative synopsis of current and prospective biomarkers of Drug induced Liver injury.

Amongst the recently explored novel DILI biomarkers in a wide range of hepatic risks include the following.

## 5 MicroRNA-122 (miRNA-122)

These are primarily involved in post-translational regulation,^[21,22]^ They represent the non-coding RNAs with remarkable specificity for hepatocytes. Indeed the liver accounts for about 75% of its total pool.^[22]^ Both mechanistic,^[22]^ and systematic studies,^[19,20]^ have demonstrated early rise in miR-122 levels even when traditional markers of liver injury such as transaminases (AST/ALT) remained normal. This early signal of hepatocyte injury demonstrated by miR-122 potentially could lend its self to utilization both as a diagnostic marker as well as risk-stratification of DILI.^[20]^ Indeed in several clinical reports, elevation of has been shown in patients with acute liver injury earlier than ALT, but crucially with no demonstrable elevation in its kinetics in comparative controls who overdosed on Acetaminophen but had no biochemical evidence of acute liver injury (rise in serum transaminases).^[23]^ Additionally, higher miR-122 levels appears to correlate with poor prognosis with studies demonstrating increasing mortality and need for liver organ support in patients with higher levels than comparative age and sex-matched controls.^[21]^ Despite these early promising reports, doubts remain regarding the exact relationship between miR-122 release, and hepatocyte injury.^[24]^ This is so because miR-122 release may be under some regulation regardless of and probably independent of hepatocyte injury. Additionally, the tendency of miR-122 for both intra and inter-individual variability in its kinetics have all combined to add to the uncertainty regarding its potential role as prospective DILI biomarker. A note of caution however remains that this is a developing field with most of the mechanistic work still evolving in non-primate species. Additionally, most of our understanding of the associations and probable predictive potential of miR-122 as a prospective DILI surrogate marker stems largely from seminal work in patients overdosed on Acetaminophen. Its role in idiopathic DILI (IDILI) still remains uncertain owing to limited quantum of studies in patients with this class of DILI phenotype. Increasing the uncertainty regarding our understanding of the diagnostic role of miR-122 is the observation from some studies that total circulating microRNA profiles may have a more robust predictive potential compared to individual mircoRNA species.^[24]^ Whatever its ultimate definitive role, microRNA estimation, and or profiling will assist in the coming years either on their own, or as composite panels with other novel liver biomarkers

## 6 Keratin-18

This is predominantly found in epithelial cells.^[7]^ It is an intermediate type-1 filament that provides cytoskeletal support to cells.^[7]^ Its full-length fragment is released in the course of hepatic cell necrosis, whereas only its Caspase-cleaved fragment gets released in the course of usual programmed cell death-apoptosis.^[7]^ The ratio between these 2 fragments therefore called the “apoptosis index” (AI) provides an indication and perhaps a magnitude of which of the 2 process (apoptosis or necrosis) is ongoing in a given index patient with suspected.^[19]^ Indeed subsequent studies exploring its role in Acetaminophen induced liver injury have reported encouraging mechanistic insight into Acetaminophen related liver injury. Crucially it rises earlier than comparative rise in serum transaminases in these studies.^[19,23]^ Indeed in the largest prospective exploration of its role in DILI to date, Dear et al^[20]^ showed that in common with other biomarkers (HMGB-1, and miR-122), full length K-18 predicted risk of acute liver compared to ALT alone in patient with Acetaminophen overdose. As has been reported with other potential DILI surrogate markers, a significant proportion of our understanding thus far has been largely due to studies (both systematic and mechanistic) in Acetaminophen induced acute liver injury.^[25]^ The totality of evidence that has accrued thus far from both observational and systematic studies suggests a determinative role in the adjudication of Acetaminophen induced acute liver injury. Further evidence in this regard will perhaps include validation of this seminal role in other patient populations.

## 7 Glutamate dehydrogenase (GLDH)

This is primarily distributed in the mitochondria of Liver, and to a lesser extent the Kidneys and skeletal muscles.^[26]^ With hepatocellular injury and loss of integrity of the mitochondrial membrane, it is released into systemic circulation with its level have been shown to rise in tandem with rise in serum ALT following Acetaminophen overdose.^[8]^ Despite a number of confounders that have been reported with its assay including issues with varying sensitivities, its consistent correlation with drug induced hepatocellular injury suggest a significant role for it as a novel DILI biomarker.

## 8 High-mobility group box-1 (HMGB-1)

This targets toll-like receptors and those associated with advanced glycation end products (RAGE).^[9]^ This chromatin-associated protein is released from necrotic cells following immune activation, and indeed a hyperglycated version of it has been associated with states of immune activation.^[27]^ Owing to the role of adaptive immune processes in the pathogenesis of DILI, there have been suggestions that kinetics of HMGB-1 may be useful both as a biomarker for DILI, as well as possible utility in providing mechanistic insight into it.^[27]^ Indeed HMGB-1 knockout mouse that have been targeted by monoclonal antibodies have been found to decrease susceptibility of developing DILI. Some isoforms of HMGB-1 have been shown to provide valuable prognostic role following Acetaminophen overdoses, including mortality and need for liver transplantation. Its selective release by necrotic and immune cells (in hyperglycated form) and not apoptotic cells suggests association with cell necrosis or immune activation. In a recent prospective study reporting on analyses of 2 clinical patient cohorts with acute liver injury (BIOPAR {*N* = 202}, and MAPP {*N* = 985}), Dear et al showed that a combined model of mirRNA-122, HMGB1, and K18 predicted risk of acute liver injury better than ALT alone (HR 1.95 [95% CI 1·87–2·03], *P* < 0.0001 in the MAPP cohort; 1.54 [1·08–2·00], *P* < .0001 in the BIOPAR cohort).^[10]^ The outcomes of these latest reports on the determinative role of HMGB-1 in DILI particularly acetaminophen induced liver injury meant the traditional algorithm of serum transaminase-based assessments are likely to change to a more novel biomarker-based strategy (with HMGB-1 perhaps at the heart of it)

## 9 Current novel biomarkers as point-of-care assays and future perspectives

### 9.1 Clinical trial and experimental drug development settings

A number of potential DILI biomarker candidates have had both FDA and European medicines agency (EMEA) approval for use in early phase drug development. This is important as it is likely to identify potential clinical drug targets with liability to cause DILI and therefore prevent the opportunity cost (ie, patient morbidities and drug development costs) that is likely to accrue were these drug related morbidities only to became apparent during the course of phase IV observation.

### 9.2 Clinical utility and future perspectives

It is evident from this review that traditional markers of liver injury despite fulfilling important roles in diagnosis of acute liver injury (of diverse etiology), have thus far proven inadequate in adjudicating cases of suspected DILI. However, despite considerable advances made in DILI candidate biomarker determinations, the evidence for their reliable and dependable utility as point-of-care assays for both diagnosis and prognostication of DILI (especially IDILI) is still accruing and will assist in their ultimate deployment as clinical supportive adjudication tools in these cohorts of patients. HMBG-1, miR-122, and K-18 in particular have recently been extensively studied by a number of well-organized systematic. The outcomes from these reports have been promising, and further validation in different populations and DILI phenotypes especially (IDILI) will only strengthen their practical utility further. The outcome of the current “omics” approach utilizing microRNA profiling, proteomics, and metabolomics to amongst others identify potential DILI-related toxicity signature molecules may likely identify reliable and dependable DILI biomarkers. There will be need for amongst others further exploration of other candidate biomarkers beyond those currently undergoing systematic evaluation. Additionally, more robust prospectively organized systematic studies in large patient populations are needed to further consolidate the diagnostic/prognostic utility of these biomarkers.

## 10 Conclusion and “take home messages”

In conclusion, traditional markers of acute liver injury such as ALT, AST, ALP TBL either employed alone or with consequential liver biopsy still remain the gold standard for the determination of various phenotypes of DILI. Prompt recognition and withdrawal of the suspected offending drug (s), as well as reporting of such reactions to relevant regulatory agencies thus far has proven clinically useful in practical management of DILI. Where etiological uncertainty exists as to the cause of Liver injury, Liver biopsy remains for now the ultimate clinical adjudicator. Potential novel markers of DILI such as HMBG-1, K-18, and miR-122 (amongst others) currently undergoing diagnostic and prognostic utility determinations are likely to revolutionarise our understanding and perhaps diagnostic strategies of DILI in years to come.

## Author contributions

**Conceptualization:** Mohammed Ibn-Mas’ud Danjuma, Jamal Sajid, Abdel-Naser Elzouki.

**Data curation:** Mohammed Ibn-Mas’ud Danjuma, Haajra fatima, Abdel-Naser Elzouki.

**Formal analysis:** Mohammed Ibn-Mas’ud Danjuma.

**Methodology:** Mohammed Ibn-Mas’ud Danjuma, Jamal Sajid, Abdel-Naser Elzouki.

**Project administration:** Haajra Fatima.

**Writing – original draft:** Mohammed Ibn-Mas’ud Danjuma, Abdel-Naser Elzouki.

**Writing – review & editing:** Mohammed Ibn-Mas’ud Danjuma, Jamal Sajid, Haajra fatima.

mohammed ibn-mas’ud danjuma orcid: 0000-0003-2198-5278.
